# Epidemiology and Clinical Impact of Pediatric Viral Gastroenteritis Before and After Rotavirus Vaccination in Sicily

**DOI:** 10.3390/pathogens15060579

**Published:** 2026-05-28

**Authors:** Floriana Bonura, Arcangelo Pepe, Dario Genovese, Emanuele Amodio, Chiara Filizzolo, Fabio Campisi, Mariangela Pizzo, Emilia Palazzotto, Simona De Grazia, Giovanni M. Giammanco

**Affiliations:** 1 Dipartimento di Promozione della Salute, Materno-Infantile, di Medicina Interna e Specialistica di Eccellenza “G. D’Alessandro”, Sezione di Microbiologia, Università degli Studi di Palermo, Via del Vespro 133, 90127 Palermo, Italy; floriana.bonura@unipa.it (F.B.); chiara.filizzolo@gmail.com (C.F.); mariangela.pizzo01@unipa.it (M.P.); giovanni.giammanco@unipa.it (G.M.G.); 2 Dipartimento di Promozione della Salute, Materno-Infantile, di Medicina Interna e Specialistica di Eccellenza “G. D’Alessandro”, Sezione di Igiene, Università degli Studi di Palermo, Via del Vespro 133, 90127 Palermo, Italy; dario.genovese@unipa.it (D.G.); emanuele.amodio@unipa.it (E.A.); 3 P.O. “G.F. Ingrassia”, Dipartimento Attività Ospedaliere, ASP 6 Palermo, C.so Calatafimi 1009, 90129 Palermo, Italy

**Keywords:** rotavirus, vaccine, rotarix, clinical severity, molecular epidemiology

## Abstract

Acute gastroenteritis (AGE) remains a leading cause of pediatric mortality and morbidity, with rotavirus as the leading cause of severe disease. Post-vaccine surveillance is essential to monitor circulating pathogens and assess vaccination impact. Sicily was the first Italian region to implement universal rotavirus vaccination in 2012. We retrospectively studied 693 children hospitalized for suspected viral AGE at the Children’s Hospital of Palermo (March 2017–February 2020), testing stool samples for viral and bacterial enteric pathogens. Rotavirus remained the most common agent (13.3%), followed by norovirus (12.1%), adenovirus (11.3%), *Salmonella* spp. (4.6%) and astrovirus (3.2%). The study population was categorized as rotavirus-associated AGE (RV-AGE) or other-cause AGE (O-AGE). Epidemiological, clinical and virological features were compared with the pre-vaccine period (2011–2012). At least one pathogen was detected in 47.5% of samples. RV-AGE cases were older than those with O-AGE (median 32.6 vs. 30.5 months; *p* < 0.01) and had greater clinical severity, with higher frequency of vomiting, fever and dehydration. Rotavirus infection was significantly associated with unvaccinated status. Compared with the pre-vaccine era, rotavirus prevalence declined (32.6% vs. 13.3%), seasonal patterns were attenuated and genotype distribution shifted toward G2P[4], G9P[8] and equine-like G3P[8] strains. Despite the decline in RV-AGE following vaccine introduction, rotavirus remains a relevant cause of pediatric AGE, underscoring the need for high vaccination coverage and continued surveillance.

## 1. Introduction

Acute gastroenteritis (AGE) remains one of the most common diseases in young children, and dehydration continues to be a leading cause of hospital admissions in industrialized countries and a significant source of mortality in developing countries. Diarrheal diseases are a major public health issue worldwide, responsible for more than 1 million deaths each year [1]. A range of microorganisms can cause diarrhea, vomiting and fever, often accompanied by abdominal pain, dehydration and headache. No single symptom can definitively differentiate viral gastroenteritis from diarrhea caused by bacterial or parasitic pathogens. Therefore, laboratory tests are necessary for identifying the causative pathogen. Enteric viruses are the leading etiological agents of AGE, with four viruses of major clinical relevance: group A rotavirus (RV), norovirus (NoV), adenovirus (AdV) types F40/41 and astrovirus (AstV) [2,3,4]. In children under five years, Rotavirus gastroenteritis (RV-AGE) remains one of the most prevalent and severe forms of AGE. Notwithstanding significant progress in prevention and treatment, diarrheal diseases continue to pose a considerable global health challenge: the latest Global Burden of Disease (GBD) estimates indicate approximately 1.17 million deaths in 2021 attributable to diarrheal diseases across all age groups, with causative agents encompassing viral and bacterial pathogens [5]. Notably, in children under 5 years, RV persists as a primary cause of lethal diarrhea (with a substantial attributable fraction among fatal cases), although its impact has diminished in regions with consistent vaccine coverage [5]. In the European Union (EU), RV-AGE puts significant pressure on healthcare systems, affecting one in every seven children under 5 years of age annually, resulting in nearly 700,000 outpatient visits, over 87,000 hospitalizations and 231 deaths [6,7,8,9]. In 2006, the World Health Organization (WHO) recommended the inclusion of RV vaccination in national immunization programs [10]. Two oral RV vaccines were licensed in 2006 and 2008 and recommended for infant immunization worldwide: the pentavalent live human–bovine reassortant vaccine RotaTeq^®^ (Merck & Co., West Point, PA, USA; PV) and the live attenuated monovalent G1P1A[8] human RVA vaccine Rotarix^®^ (GSK, Rixensart, Belgium; MV). Both vaccines are highly immunogenic, offer cross-protection against common serotypes and reduce the incidence of severe gastroenteritis, the need for intravenous fluids and hospital admissions [11]. Although RV vaccination was included in the Italian National Immunization Program only in 2017, Sicily had already pioneered its implementation by becoming the first Italian region to introduce RV vaccination with MV as part of its routine childhood immunization program in 2012. MV has been administered in Sicily using a two-dose schedule at 3 and 5 months of age. Vaccine coverage in Sicily was 6% in 2012 and progressively increased over the time, reaching 57.71% in 2024 [12]. Surveillance data indicate that the introduction of MV has substantially reduced the incidence of RV disease but also influenced the selective dynamics of strains circulating in human populations [13].

The present study aims to assess the main etiological agents in children hospitalized with AGE in Sicily in the pre- and post-vaccine era. Moreover, we analyzed the effectiveness of the MV in the local pediatric population, the seasonal distribution of enteric virus infections and the clinical outcomes in children with viral AGE. RV genotypes in the pre- and post-vaccine periods were also compared.

## 2. Materials and Methods

### 2.1. Study Population

Between March 2017 and February 2020, 693 children hospitalized for AGE at the “G. Di Cristina” Children’s Hospital in Palermo were enrolled. The hospital is one of the two main pediatric referral centers in the Palermo province, serving a pediatric population of approximately 65,000 children, as well as patients from neighboring provinces. The etiological agents of AGE were detected; the severity of all-cause gastroenteritis and the impact of MV on both RV-AGE severity and RV genotypes circulation were evaluated. Seasonal distribution of gastrointestinal pathogens and RV genotypes were compared to archived results and data from the pre-vaccine period 2011–2012. Enrolment criteria, diagnostic methods and genotyping workflows were consistent across the two study periods. Although minor changes in hospitalization policies over time may have occurred, these were unlikely to have substantially affect patient inclusion or the overall comparability between the 2011–2012 and 2017–2020 cohorts. The children were categorized into three age groups (<1 year; 1–4 years; >4 years).

### 2.2. Clinical and Anamnestic Data

For all children with AGE, informed consent was obtained, and clinical data and history (maximum number of episodes and duration of diarrhea and vomiting, fever, level of dehydration and other variables (Table 1)) were recorded, using the modified Vesikari Scale to assess the severity of gastroenteritis [14]. Other demographic data were collected through a questionnaire administered to the children’s caretakers (Table 1). RV vaccination status (vaccinated, partially vaccinated, unvaccinated) was obtained from the register of childhood vaccinations of the Sicilian region.

### 2.3. Samples Collection and Testing

A total of 693 stool samples collected from children hospitalized for AGE at the “G. Di Cristina” Children Hospital of Palermo, Italy, were tested for RV, NoV, AdV and AstV using molecular assays. Viral nucleic acids were extracted from 140 μL of 10% fecal suspensions using the QIAamp Viral RNA kit (QIAGEN, Hilden, Germany), following the manufacturer’s instructions, and reverse-transcription (RT) of RNA genomes was performed using random primers as previously described [15]. RV and NoV RNA were detected by specific real-time PCR assays targeting the NSP3 gene and the ORF1/ORF2 junction, respectively [16,17]. RV-positive samples were genotyped for G/P types through VP7 and VP4 heminested multiplex PCR reactions [18,19,20]. AstV RNA and AdV-DNA were detected using conventional RT-PCR [21,22]. Stool samples were also cultured on bacterial selective and differential media, including MacConkey agar and Salmonella–Shigella agar for the detection of *Salmonella* spp. and blood agar for the recovery of *Campylobacter* spp.

### 2.4. Statistical Analysis

Categorical data were presented as counts and percentages, whereas continuous variables were expressed as median and interquartile range (IQR) according to non-Gaussian distributions. Comparisons between RV-AGE and O-AGE were conducted utilizing Pearson’s χ^2^ test (or Fisher’s exact test when predicted cell counts were less than 5) for categorical variables, and the Mann–Whitney U test for continuous variables.

The severity of the disease was evaluated using the modified Vesikari score, classified as mild (1–5), moderate (6–9) and severe (10–18). Factors linked to escalating severity (ordinal outcome) were assessed using an ordinal adjacent-categories logistic regression model based on the proportional odds assumption, presenting adjusted odds ratios (adj OR) with 95% confidence intervals (CI). Vaccine effectiveness (VE) against RV-AGE was assessed employing a test-negative strategy, comparing vaccination status (vaccinated with at least one dose against unvaccinated) between RV-positive cases and RV-negative controls. Odds ratios (OR) were produced from multivariable logistic regression, adjusted for age in months; VE was calculated as (1 − adjusted OR) × 100 and shown as a percentage with a 95% confidence interval obtained from the confidence bounds of the adjusted OR. A *p*-value below 0.05 was deemed statistically significant. Analyses were performed using R Statistical Software (version 4.5.0).

## 3. Results

Among 693 children hospitalized between March 2017 and February 2020 for AGE, 329 (47.5%) were positive for at least one of the pathogens screened. RV was the most prevalent agent (13.3%, 92/693), followed by NoV (12.1%, 84/693), AdV (11.3%, 78/693), Salmonella spp. (4.6%, 32/693) and AstV (3.2%, 22/693). Co-infections were detected in 3% (21/693) of the samples tested and involved RV in 1.3% of cases.

**Figure 1:**
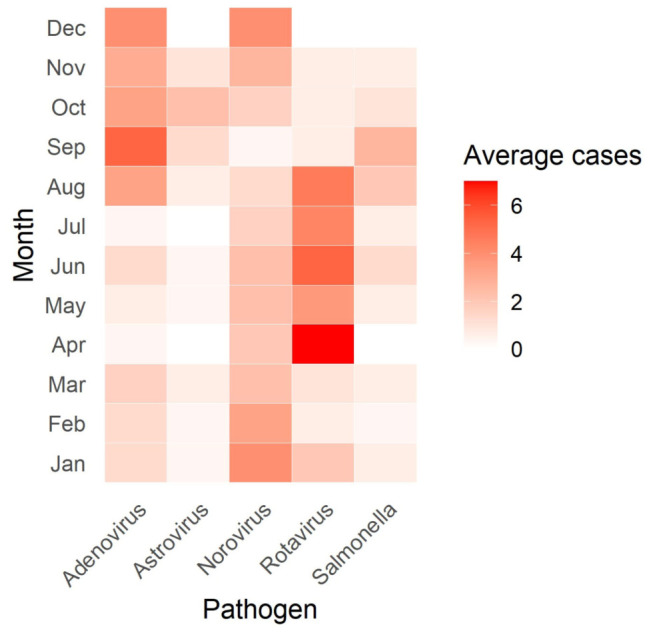
Heatmap of the seasonal distribution of gastrointestinal pathogens associated with AGE.

The heatmap analysis revealed distinct seasonal pattern in the distribution of gastrointestinal pathogens (Figure 1). In particular, RV showed a pronounced circulation during the spring and summer months, particularly in April; AdV peaked in summer and autumn, whereas NoV exhibited an increase during the winter months (from December to February). In contrast, Salmonella spp. and AstV displayed a more uniform distribution with a moderate increase observed in September and October, respectively.

The two study groups, RV-AGE and O-AGE, were compared in terms of clinical severity and other characteristics associated with their different etiologies. Statistically significant difference was observed for the median age, which was higher in RV-AGE cases (32.6 months; range 15.9–56.8) compared to O-AGE cases (30.5 months; range 13.4–63.8) (*p* < 0.01). In particular, 19.8% of RV-AGE cases were aged <1 year, 50.5% were aged 1–4 years and 29.7% were older than 4 years, while, for O-AGE cases, the proportions were 19.4%, 37.4% and 43.6%, respectively (*p* = 0.73).

Differences in anamnestic and epidemiological data between the two groups are shown in Table 1. There was no significant difference in sex distribution, and prior hospitalizations were similar across both groups, with 14.9% of RV-AGE and 12.2% of O-AGE cases reporting a history of hospitalization (*p* = 0.55). The presence of pets at home (23.2% in RV-AGE and 23.4% in O-AGE) or livestock near the home (4% in RV-AGE and 4.9% in O-AGE) was comparable between the two groups (*p* = 0.93 and *p* = 0.82, respectively). In most cases, no identifiable transmission linkage was reported, and no secondary household cases were observed. Regarding RV vaccination status, a significantly larger proportion of RV-AGE cases was in unvaccinated children (81.2%) compared to O-AGE cases (61.5%) (*p* < 0.01) (Table 1). Only 11.9% of RV-AGE cases had received two MV doses and 6.9% had received one dose, compared to 27% and 9.6%, respectively, in O-AGE cases.

RV-AGE patients had fewer days of diarrhea (median: 1 [range: 1–4]) compared to O-AGE (median: 2 [range: 1–4], *p* = 0.05), although the number of diarrheal stools was similar in both groups (RV-AGE median: 5 [range: 4–8]) vs. O-AGE (median: 5 [range: 3–8], *p* = 0.81). Vomiting was more frequent in RV-AGE (80.2%) compared to O-AGE (69.6%, *p* = 0.03), with higher median number of episodes (*p* < 0.01). A statistically significant difference was observed for fever between the two groups, with a higher frequency in RV-AGE (74.3%) compared to O-AGE (59.1%, *p* < 0.01), although maximum temperature was similar in both groups (median 38.6 °C [38.2–39] vs. 38.7 °C [38–39.3], respectively). Regardless of etiology, most children with AGE showed moderate severity of dehydration, while severe dehydration was slightly more common in RV-AGE. Median Vesikari score was higher (9 [7,8,9,10,11]) in RV-AGE compared to O-AGE (8 [6,7,8,9,10], *p* = 0.03). Mild and moderate diseases were more frequent in O-AGE (20.6% and 43.8% vs. 10,9% and 41.6%), whereas severe disease was more frequent in RV-AGE (47.5% vs. 35.6%) (*p* = 0.02) (Table 2).

For each additional month of age at hospital admission, the odds of experiencing worse severity of gastroenteritis increased by 1.008 times (95% CI: 1.003, 1.012; *p*-value < 0.01). The presence of an RV-positive stool specimen (compared to a negative test) is associated with 1.558 times higher odds of more severe gastroenteritis (95% CI: 1.144, 2.122; *p*-value < 0.01), with a significant link between RV-AGE and more severe gastroenteritis. Although children born from 2012 or later (compared to those born before 2012) show an odds ratio of 1.480 (95% CI: 0.928, 2.362), birth cohort may not have a strong effect on AGE severity (*p* = 0.1) (Table 3).

In children who did not receive the RV vaccine, the etiology of gastroenteritis was attributable to RV in 18.4% of the cases, while in the vaccinated group, the percentage of RV infections decrease to 7.7%.

To provide a more reliable estimate of vaccine effectiveness and reduce any potential bias from partially vaccinated participants, the primary analysis compared children who had completed the full vaccination schedule (two doses) with those who were unvaccinated. After adjustment for age and birth cohort (reference: birth before 2012), the odds of developing RV-AGE were substantially lower among fully vaccinated children (adjusted OR: 0.26; 95% CI: 0.14–0.50). This corresponds to an adjusted vaccine effectiveness (Adj-VE) of 74% (95% CI: 50–86), indicating a marked reduction in RV-AGE risk among children who received both doses (Table 4).

In a secondary analysis that included children who had received at least one dose, the adjusted VE was 70% (95% CI: 48–82). This suggests that meaningful protection persisted even when partially vaccinated children were considered, although their inclusion may have slightly reduced the estimated effectiveness (Table 5).

**Figure 2:**
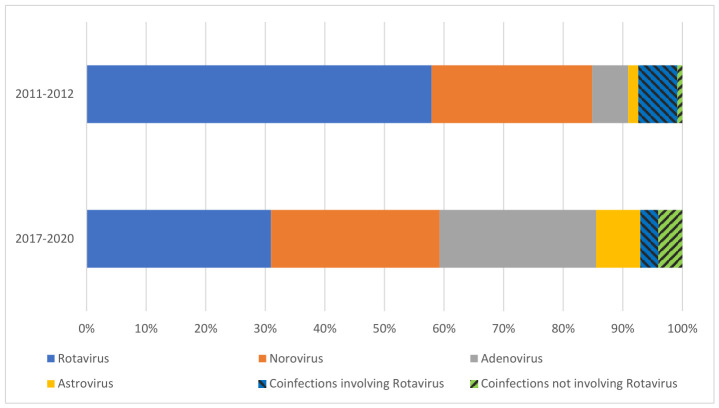
Comparison of the etiology of AGE in children hospitalized in 2011–2012 vs. 2017–2020.

Different scenarios were observed in the pre- and post-vaccine era for the etiology of viral AGE (*p* < 0.01). RV remained the most commonly identified pathogen in both eras, although its prevalence significantly decreased, from 32.6% in 2011–2012 to 13.3% in 2017–2020. NoV also decreased in percentage of cases from 15.2% to 12.1% in 2017–2020, while AdV and AstV increased (Figure 2).

Consistently with the reduction in RV infections in the post-vaccine period, also co-infections including RV significantly decreased, from 3.7% to 1.3%, while non-RV co-infections showed a slight increase (Figure 2). Details of co-infections in pre- and post-vaccine periods are shown in Table 6.

The proportion of cases with unknown agents increased from 43.8% in 2011–2012 to 52.5% in 2017–2020 (Figure 2).

**Figure 3:**
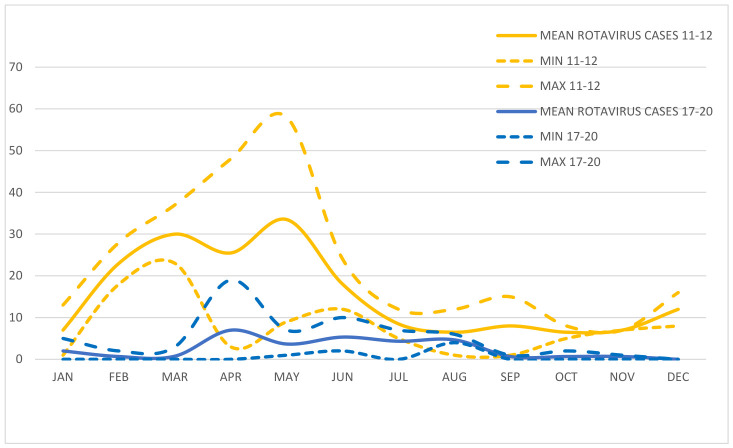
RV-AGE distribution per month in pre- and post-vaccine period (2011–2012 vs. 2017–2020). Yellow and blue solid lines represent the mean incidence during 2011–2012 and 2017–2020, respectively, while the dashed lines represent the maximum and the minimum incidence.

The average monthly incidence rate of RV-AGE in the pre-vaccine period showed a pronounced peak between February and May, confirming a late winter and spring seasonality, while in the post-vaccine period a much flatter curve due to fewer total cases was observed (Figure 3).

**Figure 4:**
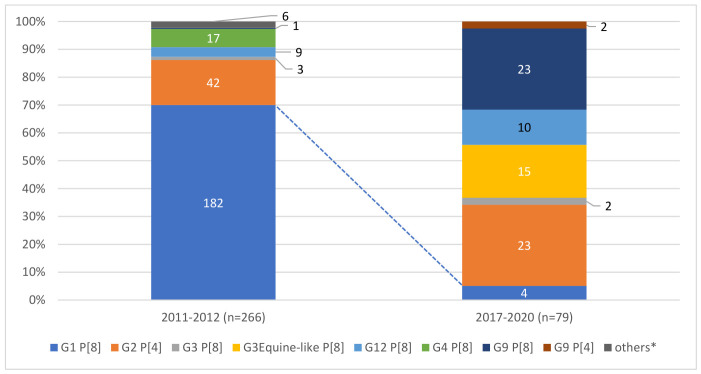
Main RV genotypes circulating in 2011–2012 vs. 2017–2020. The dotted blue line highlights the reduction in the prevalence of G1P[8] strains. * G1P[4], G2P[8], G6P[9], G3P[9], G10P[8].

A statistically significant difference was observed among RV genotypes circulating in the pre- and post-vaccine period (*p* < 0.01). In particular, in 2011–2012 the predominant genotype was G1P[8] (70.8%) followed by G2P[4] (16.3%), G4P[8] (6.6%), G12P[8] (3.5%) and G3P[8] (1.2%). All other genotypes, including G9P[8], were found to be <1%. In 2017–2020 a shift in the most common genotypes was observed, with a predominance of G2P[4] and G9P[8], each representing 29.1% of cases, while G3 equine-like P[8] emerged, accounting for 19% of infections. In contrast, G1P[8] declined considerably in prevalence, accounting for 5.1% of RV-AGE (Figure 4).

Among the 247 vaccinated children 19 tested positive for RV (7.7%). Of these, twelve had completed the full vaccination schedule, and seven (36.8%) had received only one dose, including one vaccine-strain case confirmed by sequencing (Table 7). The time since the last vaccine dose was 2.6 months (mean 16.4, range 0.2–47.7) in children who received a single dose and 22 months (mean 22.5, range 3.8–49.2) in those who completed the two-dose schedule. Disease severity, assessed by the modified Vesikari score, was lower in vaccinated RV-positive children (median 8.5, IQR 6.5) compared to unvaccinated RV-positive children (median 10, IQR 4), although this difference did not reach statistical significance (*p* = 0.15).

## 4. Discussion

Gastroenteritis remains a common cause of hospitalization among otherwise healthy children, especially within the first 5 years of life. In this study, viral enteric pathogens were detected in 47.5% of the 693 stool samples collected from hospitalized children in Palermo between March 2017 and February 2020. This overall viral detection rate is consistent with recent hospital-based molecular surveillance studies performed in Southern Italy, where approximately half of pediatric AGE admissions were attributed to viral etiologies [23,24]. Our results show that RV was the most prevalent viral pathogen, accounting for 13.3% of all AGE cases, followed by NoV, AdV and AstV. This is consistent with previous reports identifying RV as the leading viral agent responsible for gastroenteritis in children under 5 years, with NoV and AdV also recognized as prominent viral etiologies [25,26,27,28]. In our study, seasonal peaks in the incidence of AGE were observed for RV in spring–summer and for AdV in summer–autumn. In Europe, RV gastroenteritis peaks in late winter or early spring, while marked seasonality has been observed in some African countries, with a predominance of cases in the dry and relatively cold season [29]. Although hospitalizations for gastroenteritis usually reach their highest levels in the winter period [30], the seasonal distribution of each pathogen changes according to the seasonal nature of the pathogens, their cyclical emergence/re-emergence and decline, changes in environmental conditions and alterations in host behavior [31]. Although the overall incidence of RV has markedly declined following the introduction of vaccination, RV continues to exert a significant clinical burden among hospitalized children [32,33]. MV has proven effective in preventing severe RV gastroenteritis, and several European countries, including Belgium, Austria and England, observed substantial reductions in RV-associated hospitalizations after implementing universal vaccination [32,33,34]. In Sicily, after MV introduction in November 2012, a pronounced reduction in RV prevalence, from 32.6% in 2011–2012 to 13.3% in 2017–2020 was observed, concurrently with a steady rise in vaccine coverage (from 6% in 2012 to 60% in 2020). Consistently, RV infection was significantly more frequent among unvaccinated children, whereas hospitalizations among vaccinated children were predominantly attributable to O-AGE (81.2% vs. 61.5%). Our findings also suggest that vaccination may attenuate disease severity in the few breakthrough cases observed, though the limited sample size precludes definitive conclusions. These findings further underscore the critical role of vaccination in reducing both the incidence and clinical impact of RV infections.

Our vaccine effectiveness findings further support the protective impact of rotavirus vaccination in this population. When the analysis was restricted to children who had completed the two-dose schedule, the adjusted VE against RV-AGE hospitalization was 74% (95% CI: 50–86), after controlling for age at admission and birth cohort. This estimate aligns with real-world evidence on Rotarix effectiveness. In a systematic review and meta-analysis of post-licensure observational studies, the pooled VE for two doses of Rotarix was 69%, rising to 81% in high-income countries; reported variability was partly attributed to differences in study design, control selection and epidemiological context [35]. Similarly, a population-based study in Navarre, Spain, reported an adjusted VE of 78% against laboratory-confirmed rotavirus gastroenteritis and 83% against rotavirus-related hospitalization [36]. In the United States, Payne et al. estimated a VE of 80% for completion of the two-dose Rotarix schedule against rotavirus-associated hospitalizations and emergency department visits, closely overlapping with our results [37].

The slightly lower VE observed in our cohort compared with some high-income settings may reflect real-world implementation conditions in Sicily, including progressively increasing—but still moderate—vaccine coverage during the study period. Such coverage patterns may have limited indirect herd protection and could also have introduced temporal differences in vaccination probability across birth cohorts; accordingly, birth cohort was included in the final adjusted model. Notably, when children receiving at least one dose were included, VE remained substantial, although somewhat attenuated. This pattern underscores the added benefit of completing the full schedule while confirming meaningful protection under routine-use conditions.

In addition, differences in clinical severity between RV-AGE and O-AGE cases admitted to Palermo Children’s Hospital were evaluated. RV-AGE was most commonly observed in children aged 1–4 years (50.5%) and was associated with more severe gastrointestinal symptoms, including a higher frequency of vomiting, fever and profuse diarrhea. Conversely, O-AGE occurred more often in children older than 4 years (43.6%) and generally presented as a mild-to-moderate illness. These findings are consistent with previous studies indicating that RV gastroenteritis is clinically more severe than other viral etiologies, particularly in younger children, with vomiting playing a key role in dehydration and hospitalization [29,38]. Several studies have shown that the first RV infection primarily induces a homotypic serum-neutralizing antibody response, while subsequent infections elicit a broader heterotypic response [39,40]. This progressive expansion of the immune response helps explain why older children are less likely to develop severe RV gastroenteritis, as each subsequent infection broadens protection against diverse viral strains. In our study population, despite the clear clinical differences observed between the two groups, the modified Vesikari score did not differ significantly between RV-AGE and O-AGE, possibly reflecting the limited sensitivity of composite severity scores to specific symptom clusters (such as vomiting), early presentation to care or sample size-related constraints. After RV vaccine introduction the emergence or re-emergence of different genotypes has been observed. Consistent with the literature, we observed a reduction in the circulation of the dominant G1P[8] strain, with emergence of the equine like G3P[8] and an increased prevalence of G2P[4] genotype, antigenically more distant from MV strain [13,41]. However, it must be acknowledged that genotype shifts cannot be attributed exclusively to vaccine-induced immune pressure, as natural fluctuations, local outbreak dynamics and broader European epidemiological trends may also contribute to the evolving RV genotype distribution. The growing frequence of G2P[4] warrants attention, as strain diversity may affect MV performance [42]. These RV genotype dynamics reinforce the need for sustained molecular surveillance to monitor strain replacement, evaluate long term vaccine effectiveness and guide potential updates to vaccine composition. The attenuation of RV seasonality and the evolving genotype landscape underscore the dynamic epidemiology of RV in the post-vaccine era. Nevertheless, the relatively limited number of RV-positive cases may have reduced the robustness of temporal analyses, particularly those related to year-by-year and seasonal trends.

Our multivariable analysis further revealed that each additional month of age at hospital admission was associated with a modest but statistically significant increase in the odds of more severe gastroenteritis, while older age generally confers increased immunity and lower risk of severe RV gastroenteritis. RV positivity emerged as a strong predictor of severe disease, reinforcing well-established evidence identifying RV as the leading cause of severe pediatric AGE [43]. Gender did not substantially modify disease burden, although, in our cohort, RV infection was more common in boys than in girls [44].

A vaccine-associated shift in the etiological landscape of viral AGE was observed in this study, with NoV remaining the predominant pathogen and increased detection of AdV and AstV during the post-vaccine period [45,46,47,48]. The decline in RV prevalence also impacted co-infections, with cases involving RV decreasing from 3.7% to 1.3%. Therefore, vaccination may potentially mitigate disease severity associated with multi pathogen infections.

Overall, our findings indicate substantial reduction in RV burden after vaccine introduction; however, RV remains a relevant cause of severe pediatric AGE requiring hospitalization. Maintaining high vaccine uptake and ensuring continuous etiological and molecular surveillance are crucial to monitor changes in pathogen epidemiology and preserve the public health gains achieved through vaccination [49,50].

## 5. Conclusions

Our findings confirm the substantial clinical burden attributable to RV, its role as marker of disease severity and the significant benefits of vaccination in reducing RV incidence, its involvement in co-infections and RV-AGE related hospitalization. Future studies with a longer surveillance period and larger positive case numbers would help confirm and extend these observations. Maintaining high vaccination coverage remains essential to further mitigate the burden of RV-associated disease and prevent severe health outcomes in the pediatric population. Continued clinical, virological and molecular surveillance, coupled with high and equitable vaccine uptake, will be critical to sustaining these gains, anticipating shifts in pathogen prevalence and ensuring that immunization strategies remain effective against emerging strains.

## Figures and Tables

**Table 1:** Demographic and anamnestic characteristics of hospitalized rotavirus acute gastroenteritis (RV-AGE) and other agents of acute gastroenteritis (O-AGE). In bold statistically significant differences.

Variables	RV-AGE, *n* = 101	O-AGE, *n* = 592	*p* -Value
**Age at onset,** ***median months [range]***	32.6 [15.9–56.8]	30.5 [13.4–63.8]	**<0.01**
**Age at onset, ** ***n (%)***			0.73
- 0–1 years	20 (19.8%)	115 (19.4%)	
- >1–4 years	51 (50.5%)	278 (47%)	
- >4 years	30 (29.7%)	199 (33.6%)	
**Sex, ** ***n (%)***			0.71
- **Female**	44 (43.6%)	273 (46.1%)	
- **Male**	57 (56.4%)	319 (53.9%)	
**Birth cohort, ** ***n (%)***			0.28
- <2012	14 (13.9%)	112 (18.9%)	
- ≥2012	87 (86.1%)	480 (81.1%)	
**Rural areas, ** ***n (%)***			0.09
- Yes	16 (15.8%)	142 (24%)	
- No	85 (84.2%)	450 (76%)	
**Gastroenteritis transmission linkage, ** ***n (%)***			0.31
- Unknown	78 (77.2%)	469 (79.2%)	
	8 (7.9%)	28 (4.7%)	
- Contacts (Parents, Siblings, Classmates, Occasional…)	13 (12.9%)	60 (10.1%)	
	2 (2%)	24 (4.1%)	
	0 (0%)	11 (1.9%)	
**Previous hospitalizations, ** ***n (%)***			0.55
- Yes	15 (14.9%)	72 (12.2%)	
- No	86 (85.1%)	520 (87.8%)	
**Presence of pets at home, ** ***n (%)***			1
- Yes	23 (23.2%)	135 (23.3%)	
- No	76 (76.8%)	444 (76.7%)	
**Presence of livestock near the home, ** ***n (%)***			0.82
- Yes	4 (4.1%)	28 (4.9%)	
- No	94 (95.9%)	538 (95.1%)	
**Rotavirus Vaccination, ** ***n (%)***			**<0.01**
- Unvaccinated	82 (81.2%)	364 (61.5%)	
- Vaccinated with 1 dose	7 (6.9%)	60 (10.1%)	
- Vaccinated with 2 doses	12 (11.9%)	168 (28.4%)	

**Table 2:** Clinical symptoms of hospitalized rotavirus acute gastroenteritis (RV-AGE) and other agents of gastroenteritis (O-AGE). In bold statistically significant differences.

Variables	RV-AGE, *n* = 101	O-AGE, *n* = 592	*p* -Value
**Diarrhea, ** ***n (%)***	98 (97%)	569 (96.1%)	1
Number of days with diarrhea, *median [IQR]*	1 [1–4]	2 [1–4]	0.053
Number of diarrhea stools, *median [IQR]*	5 [4–8]	5 [3–8]	0.81
**Vomiting, ** ***n (%)***	81 (80.2%)	412 (69.6%)	**0.03**
Number of days with vomiting, *median [IQR]*	1 [1–2]	1 [1–2]	0.44
Number of vomiting episodes, *median [IQR]*	5 [2–10]	3 [2–6]	**<0.01**
**Fever, ** ***n (%)***	75 (74.3%)	348 (59.1%)	**<0.01**
Max recorded fever, *median [IQR]*	38.6 [38.2–39]	38.7 [38–39.3]	0.82
**Dehydration, ** ***n (%)***	87 (86.1%)	466 (78.7%)	0.09
Severity of dehydration, *n (%)*			**0.32**
- Mild	1 (1.1%)	1 (0.2%)	
- Moderate	83 (95.4%)	453 (97.2%)	
- Severe	3 (3.4%)	12 (2.6%)	
**Headache, ** ***n (%)***	11 (12.6%)	75 (15%)	0.82
**Abdominal Pain, ** ***n (%)***	54 (55.7%)	379 (65.9%)	0.06
**Modified Vesikari score ** *, ***median [IQR]***	9 [7–11]	8 [6–10]	**0.03**
Severity of disease, *n (%)*			**0.02**
- Mild	11 (10.9%)	122 (20.6%)	
- Moderate	42 (41.6%)	259 (43.8%)	
- Severe	48 (47.5%)	211 (35.6%)	

* Modified Vesikari Scale was used to assess the severity of gastroenteritis. “Mild” corresponds to a total score between 1 and 5, “Moderate” corresponds to a score between 6 and 9 and “Severe” corresponds to a total score between 10 and 18.

**Table 3:** Ordinal adjacent categories logistic regression with proportional odds assumption assessing the factors that may influence the severity of gastroenteritis (Worser ref better).

Factors Influencing AGE Severity	Adj OR (2.5, 97.5% CI) *Worser Ref Better*	*p* -Value
**Age at hospital admission (per unitary increment in months)**	1.008 (1.003, 1.012)	<0.01
**Positivity of sample for RV (ref. neg)**	1.558 (1.144, 2.122)	<0.01
**Birth cohort** ≥ **2012 (ref. < 2012)**	1.480 (0.928, 2.362)	0.1

**Table 4:** Odds ratio (OR) and vaccine effectiveness (VE) against rotavirus viral acute gastroenteritis in subjects who received two vaccine doses.

Vaccination Status	RV-AGE, *n (%)*	O-AGE, *n (%)*	Crude OR (95%)	Adj OR * (95%)	Adj-VE (95% CI)
**Unvaccinated**	82 (87.2)	364 (68.4)	Reference	Reference	
**Vaccinated (2 doses)**	12 (12.8)	168 (31.6)	0.32 (0.17, 0.6)	0.26 (0.14, 0.5)	74% (50, 86)

* Adjusted for age in months and for birth cohort.

**Table 5:** Odds ratio (OR) and vaccine effectiveness (VE) against rotavirus viral acute gastroenteritis in subjects with at least one dose of vaccine.

Vaccination Status	RV-AGE, *n (%)*	O-AGE, *n (%)*	Crude OR (95%)	Adj OR * (95%)	Adj-VE (95% CI)
**Unvaccinated**	82 (81.2)	364 (61.5)	Reference	Reference	
**Vaccinated (≥1 doses)**	19 (18.8)	228 (38.5)	0.37 (0.22, 0.63)	0.3 (0.18, 0.52)	70% (48, 82)

* Adjusted for age in months and for birth cohort.

**Table 6:** Details of co-infections in pre- and post-vaccine periods.

Co-Infections Involving RV			Co-Infections Not Involving RV		
	2011–2012 ^	2017–2020		2011–2012 ^	2017–2020
**RV + NoV**	34	3	**NoV + AdV**	2	3
**RV + AdV**	2	3	**NoV + AstV**	3	4
**RV + AstV**	2		**NoV + SS** *		2
**RV + SS** *		2	**AdV + SS** *		1
**RV + NoV + AstV**	1		**AstV + SS** *		1
**RV + NoV + AdV**		1	**SS** * **+ C** **		1

* SS = *Salmonella* spp.; ** C = *Campylobacter* spp.; ^ in the pre-vaccine era bacterial etiologies were not available.

**Table 7:** Circulating rotavirus genotypes in vaccinated patients.

Rotavirus Genotypes	*n* = 19
**G1P[nd]**	1
**G1P[8]** *	1
**G12P[8]**	1
**G3Equine-like P[8]**	2
**G9P[8]**	6
**GndP[9]**	1
**Not typeable**	7

nd: not determined. * Vaccine strain, in child with incomplete vaccination (only 1 of 2 doses).

## Data Availability

Data will be made available on request.
